# Life-Cycle Traits of *Paraleucilla magna*, a Calcareous Sponge Invasive in a Coastal Mediterranean Basin

**DOI:** 10.1371/journal.pone.0042392

**Published:** 2012-08-08

**Authors:** Caterina Longo, Carmen Pontassuglia, Giuseppe Corriero, Elda Gaino

**Affiliations:** 1 Dipartimento di Biologia, Università di Bari “Aldo Moro”, Bari, Italy; 2 Dipartimento di Biologia Cellulare e Ambientale, Università di Perugia, Perugia, Italy; McGill University, Canada

## Abstract

The calcareous sponge *Paraleucilla magna,* originally observed along the Brazilian coast (Atlantic Ocean), is the only allochthonous invasive species of Porifera reported in the Mediterranean Sea. A 1-year investigation of the population dynamics and life-cycle of this exotic species in the Mar Piccolo di Taranto (southern Italy, central Mediterranean Sea) has provided a good opportunity to test how environmental variations can influence its life-cycle and to ascertain what strategy can be adopted to successfully colonize a new environment. In the Mar Piccolo di Taranto, *P. magna* exhibits marked temporal changes in biomass. The studied specimens reproduce almost all year round, showing a seasonal pattern that peaks during warm months. This prolonged sexual activity allows *P. magna* to continuously produce young specimens, with repeated recruitment events taking place throughout the year, thus offsetting the seasonal mortality of adult specimens. This r-strategy enables the non-indigenous sponge to achieve a high degree of maintenance over relatively long periods (ten years at least).

## Introduction

The introduction of non-native species has an adverse effect on the environments colonized, in that the newcomers compete with native species for resources and space, and may carry parasites and pathogens that impact negatively the community [1], [2]. Numerous articles have dealt with the introduction of allochthonous species through human activity. Such activity may promote dispersal, causing disruption to the newly colonized ecosystems and sometimes displacing native species [3].

In marine environments, biological invasions take place through a variety of pathways of introduction, including the shipping of commercial species, live seafood shipments and vessel activity (see Occhpinti-Ambrogi et al. [4]). Protective measures have been adopted to prevent the transport of non-indigenous species and to control their spread by human vectors. However, while such measures may be highly effective in preventing the transport of some taxa, they may do little to stem the spread of others [5].

The current literature contains many reports on the invasiveness of representatives of various groups belonging to both marine, and brackish habitats [4], [6]. Increased interest in invasive fauna has led to the cataloguing of non-indigenous species, in order to assess their ecological impact on the ecosystem [7], [8]. The impact of exotic species differs from ecosystem to ecosystem, and it remains difficult to isolate mechanisms affecting their success. Anthropogenic disturbance is considered a risk factor in establishing non-indigenous species. Indeed, it has been proved that exposure to pollution reduces species diversity in hard-substrata marine communities [9], but not that of exotic components [10].

Well-known study cases of non-indigenous aquatic invertebrates are reported among molluscs [11], ascidians [3], [12], bryozoans [13], [14], polychaetes [15] and crabs [16]. Only few reports deal with non-indigenous sponges, among which particular attention has been paid to *Mycale grandis* (from the Central Pacific) and *Terpios oshinota* (from the Western Pacific), both of which compete strongly for space with native species and corals [1], [17], [18].

Habitat specificity is particularly relevant to the success of an invasive species, a feature that could be ascertained by means of long-term monitoring programs. In this regard, *Chalinula nematifera,* a species native to the Indo-Pacific region, displays an active selection of the habitat, in that it has been recorded overgrowing the live ramified corals of the genus *Pocillopora* (94% *vs* 6% on rocks) in Mexican Pacific coastal regions [19]. By contrast, when corals are invasive, as in the case of *Tubastrea coccinea* and *T. tagusensis*, the sponge *Desmapsamma anchorata* may have a positive effect on the benthic community by overgrowing and killing the coral colonies; it thus constitutes an efficient curb against the invasiveness of these species [20]. Instead, on the Indonesian coral reefs, this epizoic sponge overgrows the octocoral *Carijoa riisei*, causing a morphological adaptation, which is limited to an irregular branching pattern of the coral and in the formation a dense nematocyst layer along the contact surface of the sponge [21].

**Figure 1:**
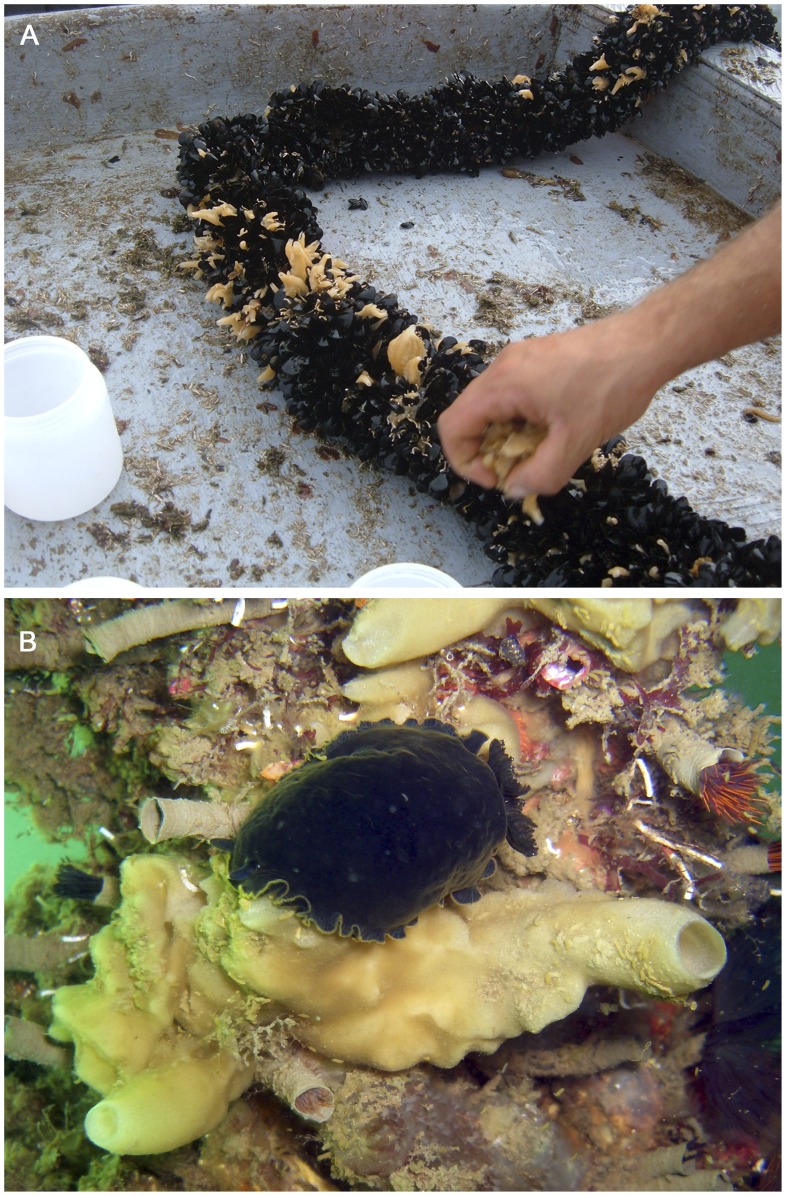
*Paraleucilla magna* in the Mar Piccolo di Taranto (central Mediterranean Sea). A) epibiontic specimens on a farmed row of *Mytilus galloprovincialis*; B) view of a large specimen inhabiting an artificial pole, submerged for several years, together with numerous tubes of the polychaete *Branchiomma luctuosum* and the nudibranch *Dendrodoris limbata.*

The Mediterranean Sea has also seen the introduction of non-indigenous species belonging to various taxa [4], [22], including a small number of sponges (see reference in Longo et al. [23], Zenetos et al. [22]). Among these the calcareous sponge *Paraleucilla magna* (Klautau, Monteiro & Borojevic, 2004) has recently been recorded in a semi-enclosed basin located in the north-western Ionian Sea (Mar Piccolo di Taranto) where it showed an invasive behaviour [23]. First identified as a new species in 2001 in the Atlantic Ocean, this sponge is very abundant along the Brazilian coast [24].

The original distribution of the genus *Paraleucilla* encompasses the Indo-Pacific region (Australian coasts) and the Red Sea (see Longo et al. [23] for detailed references). Only recently, *P. magna* has been reported in the Atlantic Ocean [24], [25] and in the Mediterranean Sea (southern coasts of Italy, Malta Island, Spain, north-western coast of the West Basin) [23], [26], [27].

The almost simultaneous finding of *P. magna* in different areas of the Mediterranean Sea and along the Atlantic coast of Brazil, makes it difficult to suppose a scenario able to describe the possible transfer of this species [23].

In spite of the unknown origin of *P. magna,* in the Mediterranean Sea this calcareous sponge is considered a non-indigenous invasive species for these reasons: i) at present, this species has been found in sites well studied in the past by sponge taxonomist who had never found it along the Italian coasts; ii) the main sites where this species has been found (Taranto, Brindisi and Naples) are relevant Mediterranean ports, with a huge volume of shipping; iii) this species has shown a rapid colonization pattern in the Central and Western Mediterranean Basin [26].

The life-cycle of *P. magna* has been investigated in the Atlantic Ocean (Rio de Janeiro, Brazil) by Lanna et al. [28] and by Lanna and Klautau [29], who also described some steps of gamete differentiation, thus proving the hermaphroditic condition of this calcareous sponge.

The aim of the present study was to investigate the life-cycle of this exotic species in a non-native environment and to ascertain how its reproductive strategy can affect the success of its colonization, thus providing useful information on the adaptive potential of this species following its finding into an introduced range and gain knowledge on its invasiveness in the Mediterranean Sea, where it constitutes the only known case of sponge invasion.

**Figure 2:**
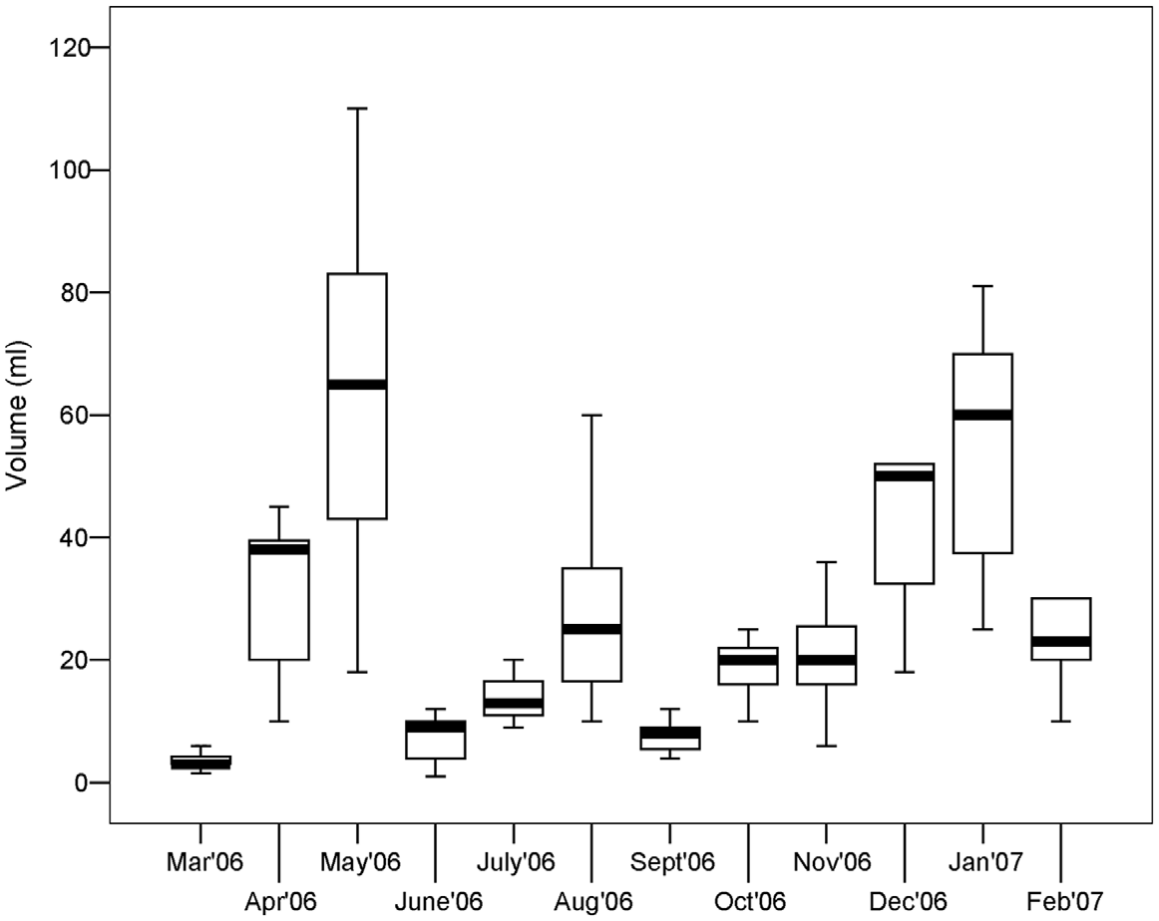
Box plots of the volume of *Paraleucilla magna* throughout the study period. Each box displays the median, upper and lower quartiles of the distribution of sponge volume per month. Box whiskers represent the maximum and minimum value.

**Figure 3:**
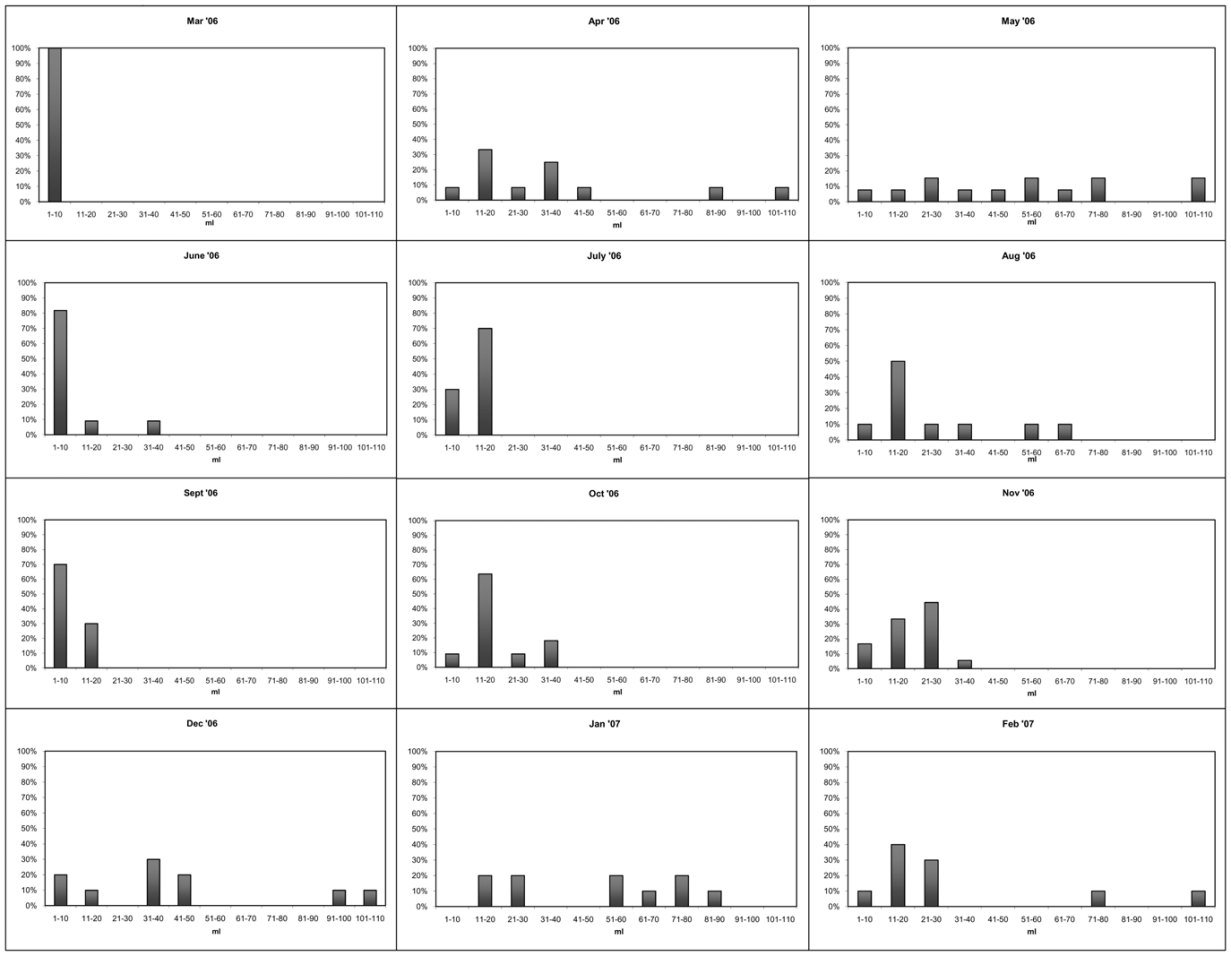
Monthly trend in the volume of *Paraleucilla magna* specimens from the Mar Piccolo di Taranto over the study period.

## Materials and Methods

### Ethics Statement

No specific permits were required for the described field studies. The sponge samples were collected from a location that is not privately-owned or protected in any way, according to the authorization of Marina Mercantile now called Ministero delle Politiche Agricole, Alimentari e Forestali (DPR 1639/68, 09/19/1980 confirmed on 01/10/2000). The Department (Dipartimento di Biologia, Università degli Studi di Bari “Aldo Moro”) has been approved by the Ministero della Marina Mercantile as an “Accredited Scientific Institution” and it is in compliance with all formalities required from “Accredited Scientific Institutions”, as reported in the Art. 27, 29, 30 of the DPR 1639/68. The field studies did not involve endangered or protected species. All animal procedures were in compliance with the guidelines of the European Union (directive 609/86).

**Figure 4:**
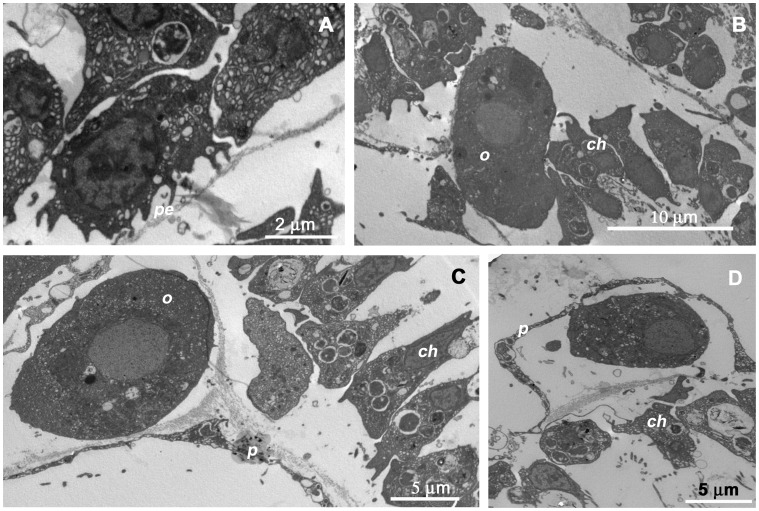
Ultrastructural details of oocytes of *Paraleucilla magna*. **A**) primordial female germ cells located below the choanoderm gifted by short pseudopodial extensions (*pe*); **B**) young oocyte (*o*) crossing the choanoderm (*ch*); **C**) oocyte interposed between the choanoderm (*ch*) and the pinacocyte-like cells (*p*); **D**) oocyte in a chamber delimited by pinacocyte (*p*) and choanocyte layers (*ch*).

Fifteen randomly selected specimens of the calcareous sponge *Paraleucilla magna* were collected monthly, at a depth ranging between 2 and 7 metres, from March 2006 to February 2007, in the Mar Piccolo di Taranto (Southern Italy); it is a semi-enclosed basin located in the north-western Ionian Sea (central Mediterranean Sea) (40°29′37.49′′N 17°15′52.71′′E), subject to strong seasonal variations of the main water ecological parameters [30], [31]. In this environment, the species is very common, living as an epibiont on mussel rows and artificial hard substrata (e.g. wooden, plastic or metal poles, floats) (Figure 1).

Soon after collection, all specimens were processed to obtain monthly estimates of volume, measured by means of a graduated cylinder. In order to investigate sponge reproduction in the study period, two 1 cm^3^ fragments were cut from each of 180 specimens and prepared for light and electron microscopy. The fragments for light microscopy were fixed in Bouin’s fixative for 4 hours; the fragments for transmission electron microscopy (TEM) were fixed in 2.5% glutaraldehyde buffered with filtered sea-water (pH = 7.8). After fixation, the fragments were rinsed with filtered sea-water and decalcified in 5% HCl for 5 hours. They were then rinsed in filtered sea-water.

For light microscopy, fragments were dehydrated in an increasing ethanol series, cleared in xylene, and embedded in paraffin. Thin sections (7 µm), processed according to the routinely used techniques for histological preparations, were mounted on glass slides and stained with toluidine blue. Sections were observed under a light microscope to determine the monthly percentage of specimens with oocytes, male elements, embryos and larvae, together with the mean diameter (considering them spherical) and the density (number/mm^3^) of reproductive elements. Gamete quantification was performed analyzing the whole histological section measured by using a reticulate cover-slip positioned above it.

**Figure 5:**
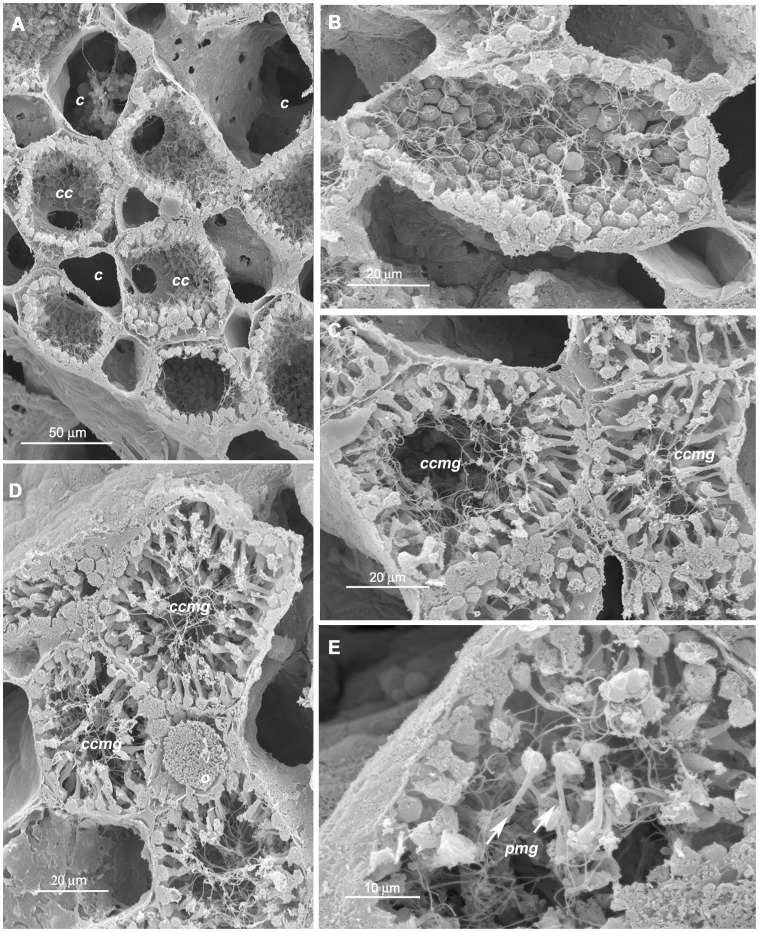
Choanocyte chambers and details of transformed choanocyte chambers with putative male gametes of *Paraleucilla magna*. **A**) choanocyte chambers (*cc*) arranged around the canals (*c*); **B**) choanocyte chamber lined by round-shaped choanocytes; **C, D**) transformed choanocyte chambers with precursor of male gametes (*ccmg*), oocyte (*o*); **E**) putative male gametes (*pmg*) directed toward the lumen of the chamber.

**Figure 6:**
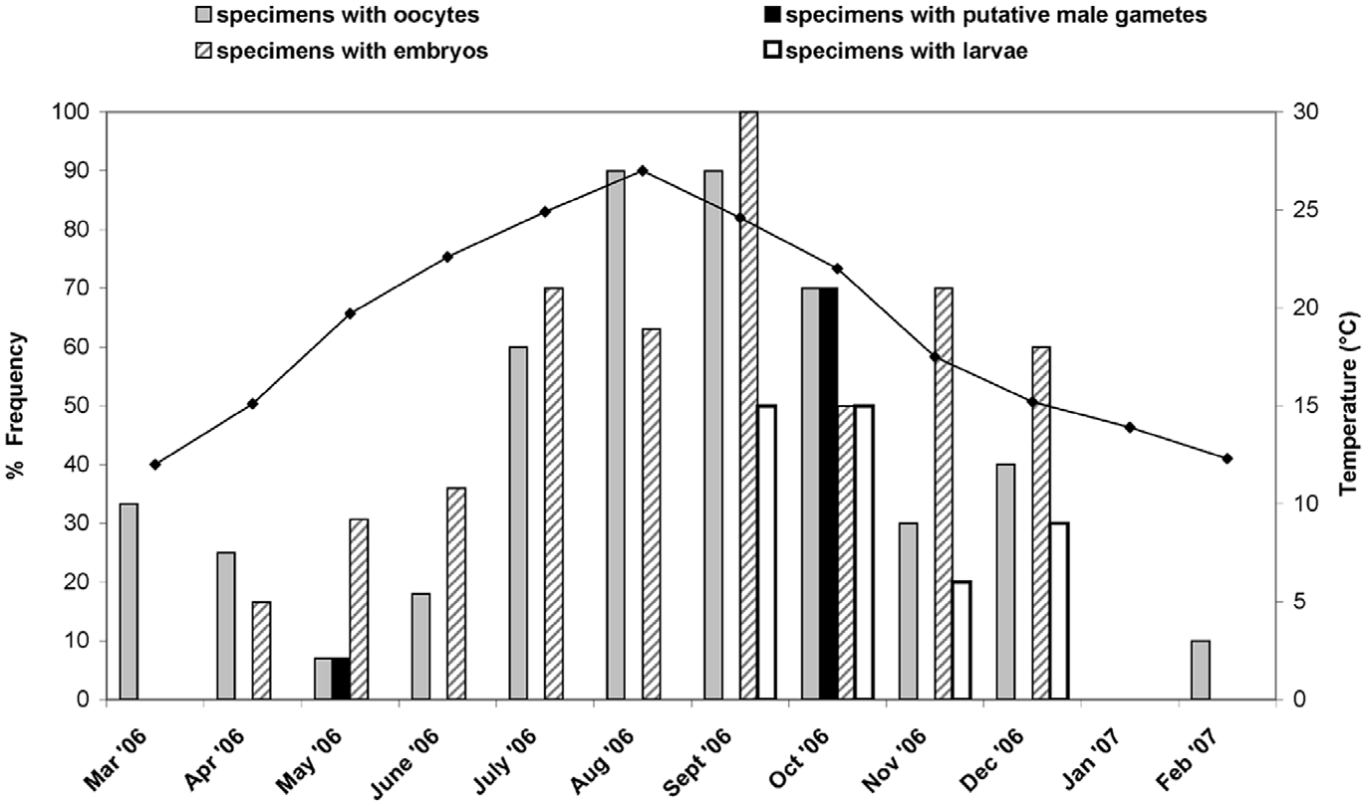
Reproductive trend of *Paraleucilla magna* throughout the year: monthly frequency of specimens with reproductive elements in relation to sea-water temperature.

The reproductive elements were quantitatively evaluated by means of the Abercrombie [32] formula, as suggested by Elvin [33].

**Figure 7:**
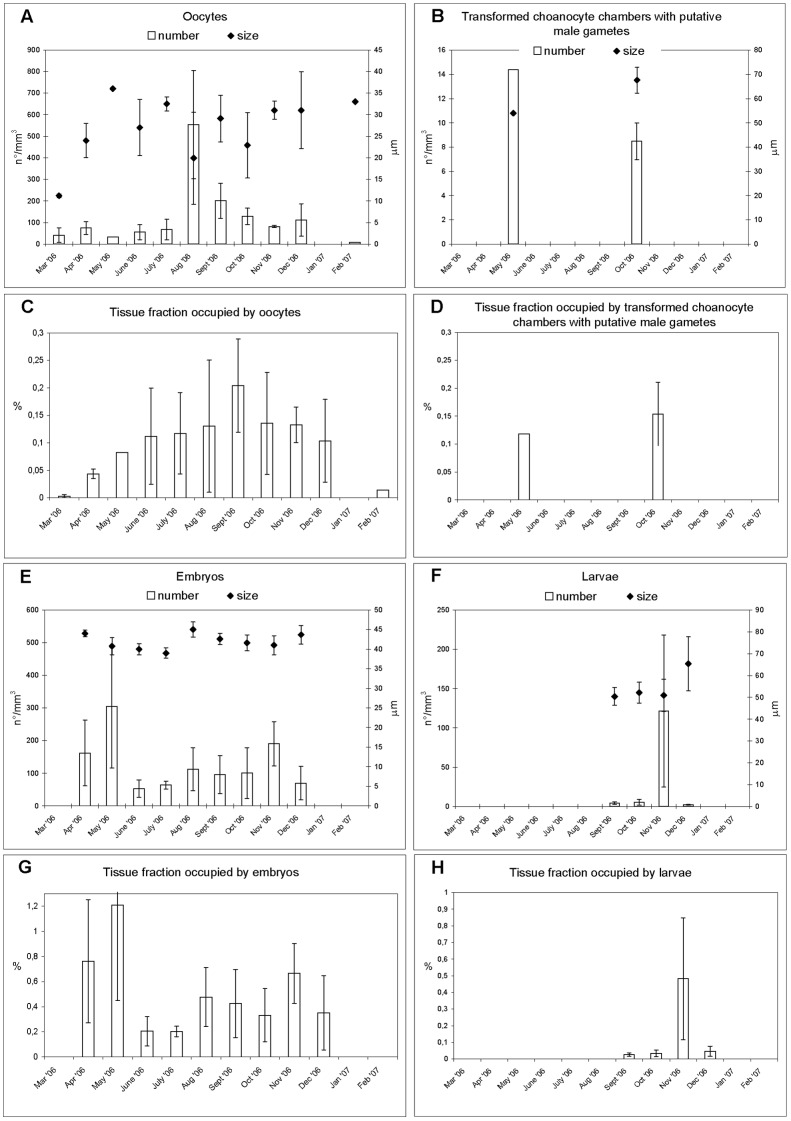
Trend in the sexual cycle of *Paraleucilla magna* in the Mar Piccolo di Taranto. Monthly mean value and standard error of size, number of reproductive elements per mm^3^ of sponge tissue (**A** = oocytes; **B** = transformed choanocyte chambers with putative male gametes; **E** = embryos; **F** = larvae) and % of sponge tissue occupied by reproductive elements (**C** = oocytes; **D** = transformed choanocyte chambers with putative male gametes; **G** = embryos; **H** = larvae).

For TEM analysis, fragments were rinsed in artificial sea-water, used as a buffer, and post-fixed in a solution of 1% osmium tetroxide in artificial sea-water for 1 hour at room temperature. Subsequently, the samples were washed in the same buffer, dehydrated in an increasing alcohol series up to propylene oxide and embedded in an Epon-Araldite mixture. Ultrathin sections were contrasted with 5% uranyl acetate for 20 minutes and lead citrate for 5 minutes, and observed under a Philips EM 208 Transmission Electron Microscope.

**Figure 8:**
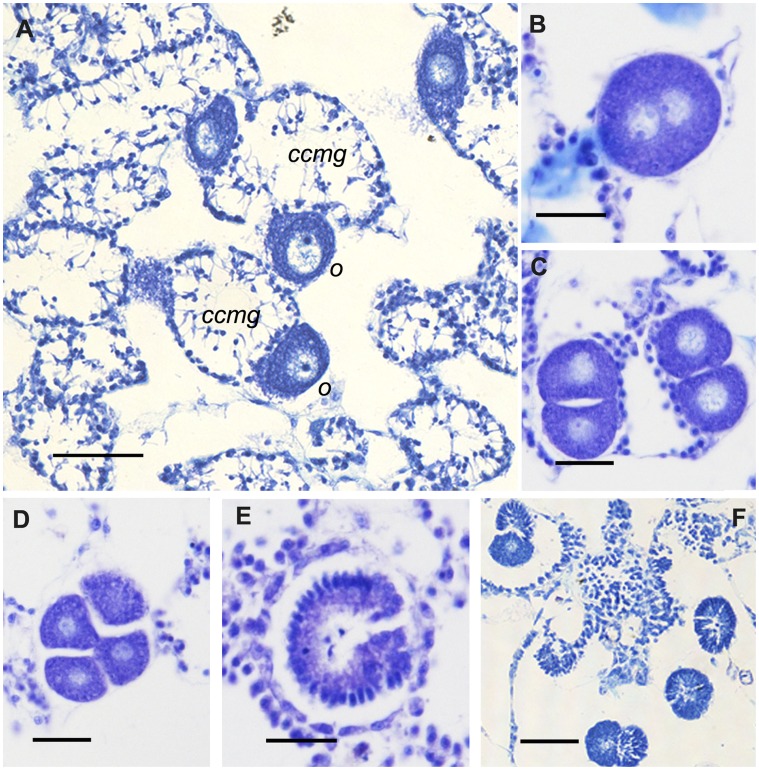
Main phases of the sexual reproductive cycle of *Paraleucilla magna*. **A**) oocytes (*o*) and transformed chonaocyte chambers with putative male gametes (*ccmg*) coexisting in the same specimen; **B**, **C, D**) segmentation process; **E**) embryo at beginning of inversion process; **F**) amphiblastula larvae (scale bars: **A**, 50 µm; **B**–**E**, 25 µm; **F**, 100 µm).

Preparations for scanning electron microscopy (SEM) were made by subjecting the paraffin-embedded material to a paraffin-dissolving xylol treatment after the removal of some sections for routine histological procedures. This technique allowed us to observe the area of tissue involved in sponge reproduction. Selected material was then critical-point dried in a CPD 030 Bal-Tec critical-point dryer (Bal-Tec, Union, Liechtenstein), mounted on stubs with silver conducting paint, sputter-coated with gold-palladium in an Emitech K550X (Emitech, Ashford, England) spatterer, and observed with a Philips XL30 electron microscope (Philips, Eindhoven, The Netherlands) at an accelerating voltage of 18 kV.

**Table 1:** The reproductive periods of *Paraleucilla magna* in the Atlantic Ocean and in the Mediterranean Sea.

	Atlantic Ocean						Mediterranean Sea					
	Brazil^1^						Southern Italy^2^			Spain^3^		
	2004	2005	2006	2007	2008	2009	2005*	2006	2007	2006	2007	2008
**Jan**	O	O	O, L	G, RC	G, RC	RC	//					
**Feb**	O, L	O, L	O, L	G, RC	G, RC	//	//	//	O			
**Mar**	O, L	O, L		G, RC	G, RC	//	//	O	//			
**Apr**	O	L	//	G, RC	RC	//	//	O, E	//	R	R	R
**May**			//	G, RC	RC	//	//	O, PMG, E	//	R	R	R
**June**		L	//	G, RC	RC	//	O, E	O, E	//	R	R	R
**July**	L		//	G, RC	RC	//	O, E	O, E	//	R	R	R
**Aug**			G	G, RC	RC	//	*//*	O, E	//			
**Sept**			G	G, RC	RC	//	O, E	O, E, L	//			
**Oct**			G	G, RC	RC	//	O, E	O, PMG, E, L	//			
**Nov**		O	G	G, RC	RC	//	O, E	O, E, L	//			
**Dec**	O	O	G	G, RC	RC	//	O	O, E, L	//			

O. oocytes; PMG. putative male gametes; E. embryos; L. larvae; G. gametogenesis;

R. reproductive period; RC. Recruits; //. data not available.

1 Lanna et al. [28], Lanna & Klautau [29], Padua et al. [36].

2 present paper, 2005*. Longo, unpublished data.

3 Guardiola et al. [27].

During the monitoring period, the water temperature was measured monthly along the water column, using a multi-parametric probe (OCEAN SEVEN 316 Plus).

With the aim to test if sponge volume differed between reproductive and non-reproductive specimens, the parametric *t*-test was performed. Moreover, to determine if sponge volume varied significantly among months the One-Way ANOVA test was performed. Finally, the relationships between reproductive parameters (number, density and percentage of sponge tissue occupied by reproductive elements) and water temperature were calculated using the Pearson correlation. When necessary, data were transformed prior to analysis. Statistical analyses were performed using SPSS for Windows version 16.0.

## Results

The external morphology of the specimens of *Paraleucilla magna* collected from the Mar Piccolo di Taranto differed markedly within the same sampling period. No relationship was detected between the shape of the sponge and the presence of reproductive elements. Most of the specimens collected had a massive body with a smooth surface and numerous oscula. The colour of live specimens varied from beige to light yellow (Figure 1).

*P. magna* exhibited marked temporal variations in biomass (Figure 2). A high monthly variability showed two peaks in April–May 2006 and December 2006–January 2007, with a trough in between.

Analysis of the size distribution of *P. magna* revealed that in some months (March, July, September 2006), the largest specimens were absent and only young sponges attributable to the smallest classes were found (Figure 3). Monthly differences in sponge volume were significant at the statistical analysis (F_11,168_ = 17.6, p<0.001). The volume measurements of the 180 collected specimens revealed that population size distribution was characterized by an abundance of small sponges.

### Reproductive Specimens

The sexual reproduction of *P. magna* has recently been investigated by Lanna and Klautau [29], who described gamete differentiation in specimens from Brazil. At the ultrastructural level, the images of the specimens from the Mar Piccolo di Taranto integrate previous descriptions of the gametes of this species. According to Lanna and Klautau [29], primordial female germ cells originate from choanocytes which have lost their collar and flagellum. In this regard, sections of the specimens from the Mar Piccolo di Taranto showed the presence of cells located below the choanoderm and characterized by short pseudopodial extensions, a feature consistent with an initial translocation (Figure 4A). This locomotory activity is essential for the young oogonia derived from choanocytes to leave the choanocyte chamber for their ensuing growth. In fact, the presumed young oocytes were observed crossing the choanoderm (Figure 4B) to enter the mesohyl. Oocytes, became interposed between the choanoderm and the pinacocyte-like cells (Figure 4C), in such a way that the further vitellogenic growth took place in a chamber delimited by pinacocyte and choanocyte layers (Figure 4D).

The choanoderm was also involved in male gamete differentiation. In fact, whereas the typical choanocyte chambers were arranged around the canals (Figure 5A) and lined by round-shaped choanocytes (Figure 5B), some other chambers differed from the previous ones because they were delimited by cells whose location was coherent with their belonging to transformed choanocytes (Figure 5C). These cells consisted of two distinct parts: a basal round portion adherent to the chamber wall and a thin portion protruding towards the centre of the chamber (Figure 5C, D). Some cells, after detaching from the chamber wall, tended to gather in the lumen of the chamber (Figure 5E). Oocytes were evident among the transformed chambers (Figure 5D), thus supporting the hermaphroditic condition of this species.

With the exception of January, in the remaining months the specimens displayed reproductive elements (Figure 6). The monthly frequency of reproductive specimens ranged from a minimum in winter-spring to a maximum in summer-autumn. Oocytes were observed all year round. Transformed choanocyte chambers with putative male gametes were present only in May and October. Embryos occurred from April to December, with a peak in September; larvae were detected from September to December (Figure 6). In October, all types of reproductive elements were observed (Figure 6). In particular, in this month, oocytes and transformed choanocyte chambers with putative male gametes coexisted in the same specimen.

Oocytes (Figure 7) showed their minimum dimension of 10 µm in March. In May, at the end of vitellogenic growth, they reached the largest dimension (37 µm), concomitantly with the maximum density of transformed choanocyte chambers with putative male gametes (14.4/mm^3^). On calculating the percentage of tissue containing reproductive elements and transformed choanocyte chambers (0.20±0.08%), it emerged that oocytes reached their maximum density values (553.6±250/mm^3^) in August and September (Figure 7).

Fertilized eggs showed total and equal cleavage (Figure 8). The final result of this process was a stomoblastula-like embryo, evident from April to December, which reached its maximum size in August (45±1.9 µm). The inversion process led to the amphiblastula larvae, which reached their maximum size and density in December and November (62.33±12.38 µm and 121.56±96.78/mm^3^, respectively).

On considering the pattern of reproduction in relation to the water temperature values (Figure 6), it emerged that the increase in specimens with oocytes took place in parallel with the temperature, the maximum value of which coincided with the highest number of specimens carrying oocytes. The frequency of specimens with embryos increased and decreased concomitantly with the increase and decrease of the temperature values. The statistical analysis showed a significant positive relationships between the percentage of tissue involved in sexual reproduction and water temperature only for oocytes (Pearson coefficient of correlation r = 0.77, p<0.01).

## Discussion

Sponge species can be transferred from one ocean to another via human-mediated transport [34], a dispersal mechanism that has recently been proposed for a north-western Pacific sponge that was introduced into the north-eastern Atlantic together with a Pacific oyster [35]. A similar dispersal mechanism has been hypothesized for *Paraleucilla magna* [23], whose members have occasionally been introduced into Mediterranean Sea through aquaculture activity. A recent record in Malta, where this species was found as a component of fouling community located around fish-farms [26], seems to confirm this scenario.

So far, this calcareous sponge has been reported in a large portion of the western Mediterranean, i.e.: north-western Ionian Sea, southern Adriatic Sea, central Tyrrhenian Sea, Spanish coast (Blanes), central Mediterranean (Malta) and north-western Mediterranean coasts [23], [26], [27]. The first record of the presence of this sponge in the Mediterranean was in 2001, when Longo et al. [23] reported the occurrence of rich benthic assemblages of filter-feeders, characterized by large numbers of specimens of *P. magna*, in the Mar Piccolo di Taranto. At present, after more than ten years from the first report, this species is still very abundant in the Mar Piccolo (Corriero, unpublished observations).

The availability of biological data on the invasive sponge *P. magna* in the Mar Piccolo di Taranto, the Iberian Peninsula and Rio de Janeiro provides a good opportunity to test how environmental variations can influence the life-cycle of this species and to ascertain what strategy this species can adopt to successfully colonize a new environment.

With regard to *P. magna* from Rio de Janeiro, the reproductive period prevalently took place during summer (from January to March) [28] (Table 1), even though in more recent studies recruits of the same species were continuously found on artificial panels over 2 years of experiment [36] and reproductive specimens were detected all year round [29]. In particular, oocytes became larger (20–30 µm) and more abundant in February, when embryos in different developmental stages and larvae were found. In March, the number of reproductive elements decreased and larvae became more abundant than oocytes. Some isolated reproductive events were observed in June, but only in a small number of specimens [29]. A summer reproductive pattern prevails among the Calcarea sponges in the northern hemisphere [37], [38], [39], [40], [41], [42], with the exception of *Petrobiona massiliana* in which sexual reproduction occurs for more than half of the year (April–October) [43].

In the present study, the observation of transformed choanocyte chambers made up of cells whose morphology differs from that of the typical choanocytes is coherent with their belonging to male gametes, a feature that corroborates previous data presented by Lanna and Klautau [29] for *P. magna*. They first observed that spermatogenesis occurred in the lumen of differentiated choanocyte chambers, without the formation of actual spermatic cysts. Hourglass-shaped primordial germ cells, like those observed in the specimens from the Mar Piccolo di Taranto, are the precursors of the ensuing spherical spermatogonia. Similarly, modified choanocytes of *P. massiliana* were hypothesized to correspond to a stage in the formation of spermatogonia [43].

In the Mar Piccolo *P. magna* reproduces all year round (Table 1). In fact, some specimens had already oocytes in early spring, along with embryos and larvae, which coexisted until December. The spring–summer increase of water temperature seems to be the key factor that triggers reproduction, likewise in many Mediterranean sponges [44], [45], [46]. In particular, the seasonal variation in water temperature usually exceeds 15°C, ranging from 10–12°C in winter to almost 30°C in summer. Here, the number of specimens carrying oocytes increases concomitantly with water temperature (Pearson coefficient of correlation r = 0.72, p<0.01), and the density of oocytes reaches its maximum in parallel with the highest temperature values (Pearson coefficient of correlation r = 0.64, p<0.05). Large annual changes in water temperature may affect sponge sexual reproduction within a temperature range varying according to the geographical distribution of the species [47]. The occurrence of a summer peak in sexual reproduction (concomitantly with water temperature values around 28–30°C), is well known in many demosponges and calcareous sponges from temperate and subtropical areas [38], [48], thus supporting the hypothesis of a warm-water origin of *P. magna*. The occurrence of specimens with embryos and larvae over a long period supports the hypothesis that the phases of larval release and recruitment are long-lasting events. This hypothesis is coherent with sponge size distribution, which reveals the occurrence of the smallest size-classes for most of the study period.

In Rio de Janeiro, the pattern of reproduction in spring-summer did not seem to be linked with the seasonal variation in water temperature, as the onset of reproduction could presumably be due to the increase in primary production [28].

In the Mar Piccolo, the sponge exhibits a high monthly variability in biomass values, with two seasonal peaks (spring and winter months) separated by a drop, a trend similar to that previously observed by Longo et al. [23] in the same sponge population. This drop in the summer biomass values may be due to massive mortality among adult specimens, which tend to detach from the substratum when they reach their largest sizes, owing to the fragility of their tissues. Indeed, in the Mar Piccolo, the habitat of this sponge mainly consists of vertically-oriented artificial substrata. This hypothesis is supported by several reports of large specimens lying on the soft bottom of the basin (Corriero and Longo, unpublished observations). Marked variations in sponge biomass are also reported for the specimens of *P. magna* inhabiting the Brazilian coast, where the mean size of this sponge varies significantly throughout the year [28].

Our data are also in agreement with recent observations by Guardiola et al. [27] concerning a western Mediterranean population of *P. magna* (Table 1), in which large individuals disappear after larval release (in early summer), although the population persists throughout the year since it is replenished by recruits resulting from the same released larvae. However, on the basis of an exhaustive analysis encompassing spatial genetic differentiation and structure, clonality, and temporal differentiation in three close populations of *P. magna* from the NE of the Iberian Peninsula, the authors pointed out that the species is a good opportunistic colonizer but highly sensitive to stochastic events affecting recruitment. This suggests a high impact of the species in favourable habitats (sea culture and sheltered zones) and a low-medium influence in native communities [27]. In the Mar Piccolo di Taranto, it is reasonable to hypothesize that the prolonged sexual activity allows *P. magna* to continuously produce young specimens, with repeated recruitment events taking place throughout the year. This feature is able to offset the seasonal mortality of adult specimens. Differently from what reported for the Iberian populations, here *P. magna* is able to interfere with indigenous species in the process of colonization of hard substrates [49] and large patches of sponge specimens often overgrow other benthic organisms as ascidians and bryozoans, but also living bivalve shells, sometime becoming a problem for local mussel farmers [23] (Corriero and Longo, unpublished observations).

Similarly to what observed at Rio de Janeiro [28], sexual reproduction in *P. magna* from Taranto is not size dependent, as confirmed by *t*-test (p>0.05) performed to verify if sponge volume differed between reproductive and non-reproductive specimens. Oocytes already occur in new recruits (March), and the highest frequency of specimens with embryos and larvae has been detected in September, when all the collected specimens showed low size values. Even though in Porifera the first sexual elements may also appear in small young specimens [50], in several species sexual reproduction seems to be usually limited to individuals above a certain minimal size or age [47]. The traits here observed confirm that, in this new colonized environment, *P. magna* has adopted an r-strategy, which allows this non-indigenous sponge to achieve a high degree of maintenance over relatively long periods (ten years at least).
